# Incidence trends and survival analysis of appendiceal tumors in the United States: Primarily changes in appendiceal neuroendocrine tumors

**DOI:** 10.1371/journal.pone.0294153

**Published:** 2023-11-13

**Authors:** Dan Wang, Heming Ge, Yebin Lu, Xuejun Gong

**Affiliations:** 1 Department of General Surgery, Xiangya Hospital, Central South University, Changsha, China; 2 National Clinical Research Center for Geriatric Disorders, Xiangya Hospital, Central South University, Changsha, China; 3 Department of General Visceral and Thoracic Surgery, University Medical Center Hamburg-Eppendorf, Hamburg, Germany; University of Turin, S. Anna Hospital, ITALY

## Abstract

**Background:**

Appendiceal tumors are considered to be a relatively rare tumor of the gastrointestinal tract and the prognosis is unclear. This study comprehensively investigated trends in the epidemiology and survival of appendiceal tumors in the United States over the past approximately 20 years.

**Methods:**

Patients with pathologically confirmed appendiceal tumors from 2000 to 2017 were selected from the Surveillance, Epidemiology and End Results (SEER) database. Age-adjusted incidence rates were calculated by SEER*Stat 8.4.0. The Kaplan-Meier method was used to analyze survival and prognostic factors were investigated by a multivariate Cox proportional risk model.

**Results:**

Ultimately, 13,546 patients with appendiceal tumors between 2000 and 2017 were included. The annual incidence of colonic adenocarcinoma and mucinous adenocarcinoma remained relatively stable. Interestingly, the annual incidence of appendiceal neuroendocrine tumors (aNETs) increased significantly, from 0.03 to 0.90 per 100,000 person-years, with the most dramatic increase in the number of patients with localized disease. Patients with aNETs showed a significant improvement in survival between 2009–2017, compared to the period 2000–2008. Moreover, this improvement in survival over time was seen at all stages (localized, regional, distant) of aNETs. However, this improved survival over time was not seen in colonic and mucinous adenocarcinoma.

**Conclusions:**

The incidence of appendiceal neoplasms has increased significantly over the past nearly two decades, which is mainly due to the increased incidence and significant migration to earlier stages in aNETs. We must note that despite the increased incidence of aNETs, survival rates have improved at different disease stages.

## Introduction

Primary appendiceal tumor is extremely rare in clinical practice, accounting for only 4% of intestinal tumors, but should not be overlooked because of its special nature [1]. First of all, malignant appendiceal tumors often present as signs of acute appendicitis and are diagnosed incidentally during pathological examination of surgical specimens [2]. Appendiceal tumor may also be asymptomatic and be detected incidentally during a colonoscopy or imaging study. When patients present with significant symptoms such as abdominal distention and pain, this often indicates advanced disease development, such as peritoneal dissemination [3]. This insidious pattern of onset is one of the reasons contributing to the poor prognosis of appendiceal tumor.

In addition, the diversity of pathological types of appendiceal tumor, including colonic adenocarcinoma, mucinous adenocarcinoma and neuroendocrine tumor, further increases the difficulty in the management of the disease [2, 4]. Although adenocarcinoma is the main pathological type of appendiceal tumor, neuroendocrine tumors (aNETs) are receiving increasing attention due to their different grading, staging and better prognosis [5]. Moreover, these three pathological types differ significantly in their biological behavior. Observational studies have shown that the natural history of neuroendocrine tumors is relatively slow, with only a few developing extensive disease [6]. With advances in medical technology, the overall survival of cancer worldwide has improved significantly from two decades ago, and the incidence of certain tumors has declined [7]. However, the incidence and survival of various types of appendiceal tumors, especially aNETs, have been unknown for the past 20 years. A more updated and focused study on the incidence and survival trends of appendiceal tumors at different stages would contribute to a better understanding of these diseases.

The purpose of this study was to analyze trends in the survival and incidence of appendiceal tumor in the United States over the last 20 years through the Surveillance, Epidemiology and End Results (SEER) cancer registry.

## Materials and methods

### Patient selection

On July 28, 2022, the study cohort was extracted from the SEER 18 Registry custom database, which covers approximately 30% of the U.S. population and represents most races in the United States [8]. This analysis included patients with pathologically diagnosed appendiceal tumors between 2000 and 2017, with pathological types including colonic adenocarcinoma, mucinous adenocarcinoma and aNETs. The following cases were excluded from this study: patients without pathological confirmation, patients with a survival time of 0 months or unknown. Patients were grouped by year of diagnosis (2000–2008, 2009–2017) for demographic and clinical characteristics, including race, age, gender, marital status, tumor grade (I, II, III/IV), summary stage (localized, regional, distant) and treatment information (surgery and chemotherapy). With reference to previous studies, Grade III and Grade IV were reclassified into one category for the analysis [9, 10]. Localized stage means that the tumor is confined to the appendix (T1-3) and there is no metastasis to lymph nodes or other organs or tissues. Regional stage is defined as tumor tissue breaking through the plasma membrane layer to invade adjacent tissues/organ (T4), or regional lymph node metastasis. Distant stage refers to the presence of distant metastases [11].

This study was a retrospective analysis based on information from the SEER database and no identifiable patient information was used. Therefore, written informed consent was not required for this study and ethics committee approval was not necessary. The study was based on the ethical standards of the Declaration of Helsinki and complied with national and international norms.

### Statistical analysis

Age-adjusted incidence rates (in 1-year increments) were calculated by year of diagnosis via SEER*Stat 8.4.0 and plotted accordingly. The Log-rank test and Kaplan-Meier (K-M) method were used for survival analysis and plotting of survival curves, respectively. Using GraphPad Prism 8, the overall survival (OS) and disease-specific survival (DSS) were compared separately for different stages, grades, and years of diagnosis. Finally, the association between the included factors and OS was analyzed by SPSS 25.0 software (IBM, Armonk, NY, USA) using univariate and multivariate Cox proportional risk regression models. All tests in the analysis were two-side and p-values less than 0.05 were considered statistically significant.

## Results

### Demographics

A total of 13,546 patients with appendiceal tumors from 2000 to 2017 were extracted from the SEER database. Stratified by type of pathology, 5013 (37.01%) cases were colonic adenocarcinomas, 4833 (35.68%) cases were mucinous adenocarcinomas and 3700 (27.31%) cases were aNETs. Approximately 83.60% of the patients were white, 74.86% were over 45 years of age and 68.83% had no distant metastases at the time of diagnosis (**Table 1**). Most patients with aNETs were under 45 years of age (2036/3700, 55.03%), white (3203/3700, 86.57%) and with non-metastatic disease at the time of diagnosis (3521/3700, 95.16%). Tumor grade was available in 2888 (78.05%) of 3700 patients with aNETs, with grade I tumors being the most frequent (2525/2888, 87.43%). For treatment, 98.51% of patients with aNETs underwent surgery and 98.00% of aNETs patients did not receive chemotherapy (**Table 2**).

**Table 1:** Baseline characteristics of the all appendiceal tumor patients.

Characteristic	Year of diagnosis		p-value	Total (N = 13546)
	2000–2008 (N = 4133)	2009–2017 (N = 9413)		
Histology			<0.001	
Adenocarcinoma	1862(45.05%)	3151(33.47%)		5013(37.01%)
Mucous adenocarcinoma	1923(46.53%)	2910(30.92%)		4833(35.68%)
aNETs	348(8.42%)	3352(35.61%)		3700(27.31%)
Sex			0.046	
Female	2218(53.67%)	5226(55.52%)		7444(54.95%)
Male	1915(46.33%)	4187(44.48%)		6102(45.05%)
Race			0.012	
White	3501(84.71%)	7824(83.12%)		11325(83.60%)
Black	393(9.51%)	917(9.74%)		1310(9.67%)
Other and Unknown	239(5.78%)	672(7.14%)		911(6.73%)
Marital status			<0.001	
Married	2528(61.67%)	5017(53.30%)		7545(55.70%)
Single	1469(35.54%)	3863(41.04%)		5332(39.36%)
Unknown	136(3.29%)	533(5.66%)		669(4.94%)
Age			<0.001	
44 years or younger	803(19.43%)	2603(27.65%)		3406(25.14%)
45 to 60 years	1504(36.39%)	2948(31.32%)		4452(32.87%)
60 years or older	1826(44.18%)	3862(41.03%)		5688(41.99%)
Grade			<0.001	
I	781(18.90%)	3871(41.12%)		4652(34.34%)
II	981(23.74%)	2272(24.14%)		3253(24.02%)
III/IV	570(13.79%)	1244(13.22%)		1814(13.39%)
Unknown	1801(43.57%)	2026(21.52%)		3827(28.25%)
Stage			<0.001	
Localized	1408(34.07%)	4495(47.75%)		5903(43.58%)
Regional	1183(28.62%)	2238(23.78%)		3421(25.25%)
Distant	1425(34.48%)	2515(26.72%)		3940(29.09%)
Unknown	117(2.83%)	165(1.75%)		282(2.08%)
Surgery			0.665	
No	224(5.42%)	527(5.60%)		751(5.54%)
Yes	3909(94.58%)	8876(94.40%)		12795(94.46%)
Chemotherapy			<0.001	
No	2753(66.61%)	6582(69.92%)		9335(68.91%)
Yes	1380(33.39%)	2831(30.08%)		4211(31.09%)

**Table 2:** Baseline characteristics of the aNETs.

Characteristic	Year of diagnosis		p-value	Total (N = 3700)
	2000–2008 (N = 348)	2009–2017 (N = 3352)		
Sex			0.203	
Female	226(64.94%)	2060(61.46%)		2286(61.78%)
Male	122(35.06%)	1292(38.54%)		1414(38.22%)
Race			0.049	
White	311(89.37%)	2892(86.28%)		3203(86.57%)
Black	26(7.47%)	247(7.37%)		273(7.38%)
Other and Unknown	11(3.16%)	213(6.35%)		224(6.05%)
Marital status			0.004	
Married	153(43.97%)	1704(50.84%)		1857(50.19%)
Single	178(51.15%)	1414(42.18%)		1592(43.03%)
Unknown	17(4.88%)	234(6.98%)		251(6.78%)
Age			0.089	
44 years or younger	173(49.71%)	1863(55.58%)		2036(55.03%)
45 to 60 years	97(27.87%)	787(23.48%)		884(23.89%)
60 years or older	78(22.42%)	702(20.94%)		780(21.08%)
Grade			<0.001	
I	66(18.97%)	2459(73.36%)		2525(68.24%)
II	11(3.16%)	309(9.22%)		320(8.65%)
III/IV	10(2.87%)	33(0.98%)		43(1.16%)
Unknown	261(75.00%)	551(16.44%)		812(21.95%)
Stage			<0.001	
Localized	170(48.85%)	2738(81.68%)		2908(78.59%)
Regional	118(33.91%)	495(14.77%)		613(16.57%)
Distant	42(12.07%)	67(2.00%)		109(2.95%)
Unknown	18(5.17%)	52(1.55%)		70(1.89%)
Surgery			0.007	
No	11(3.16%)	44(1.31%)		55(1.49%)
Yes	337(96.84%)	3308(98.69%)		3645(98.51%)
Chemotherapy			<0.001	
No	331(95.11%)	3295(98.30%)		3626(98.00%)
Yes	17(4.89%)	57(1.70%)		74(2.00%)

### Trends in incidence over time

**Fig 1:**
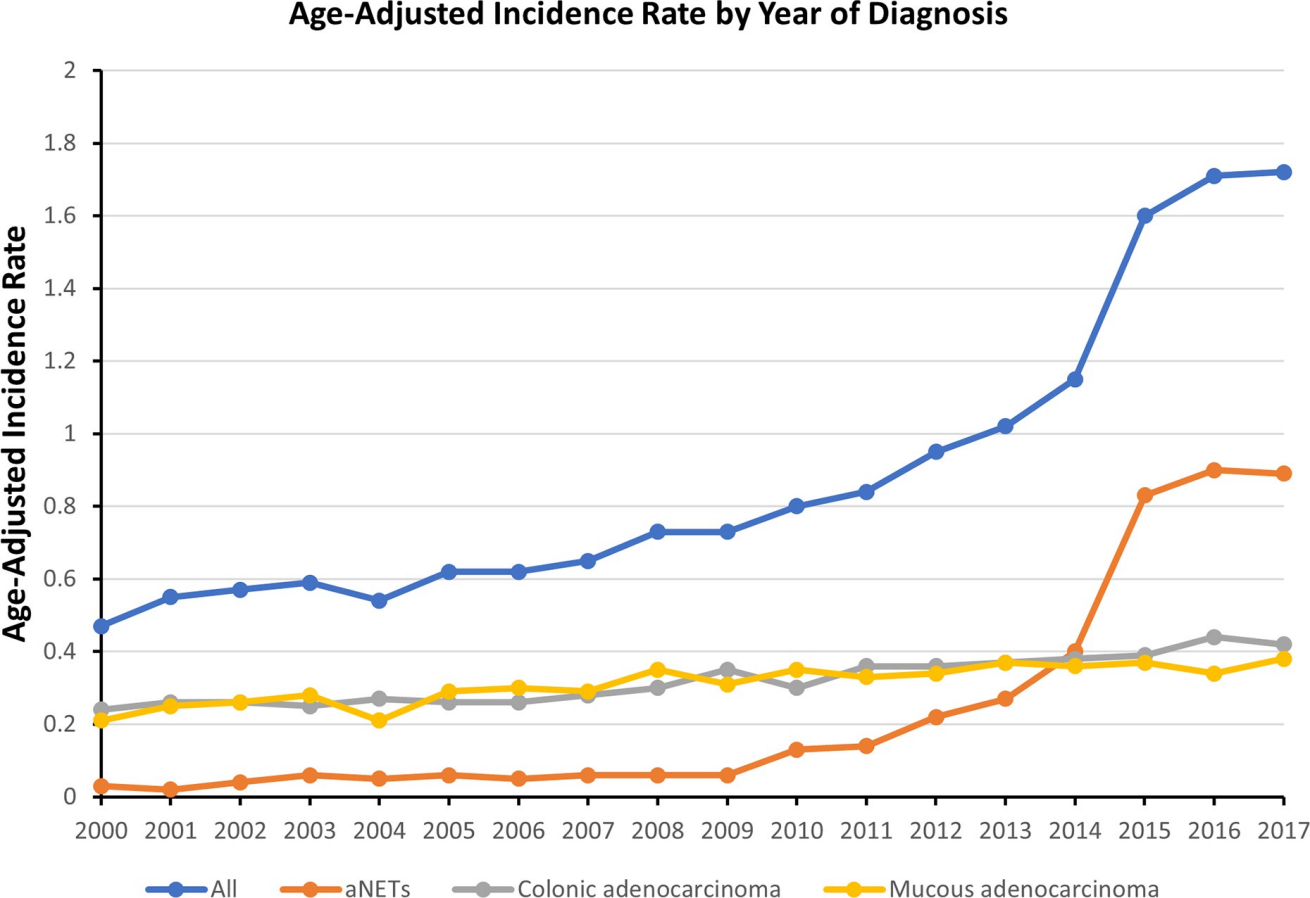
Age-adjusted incidence rate by year of diagnosis.

**Fig 2:**
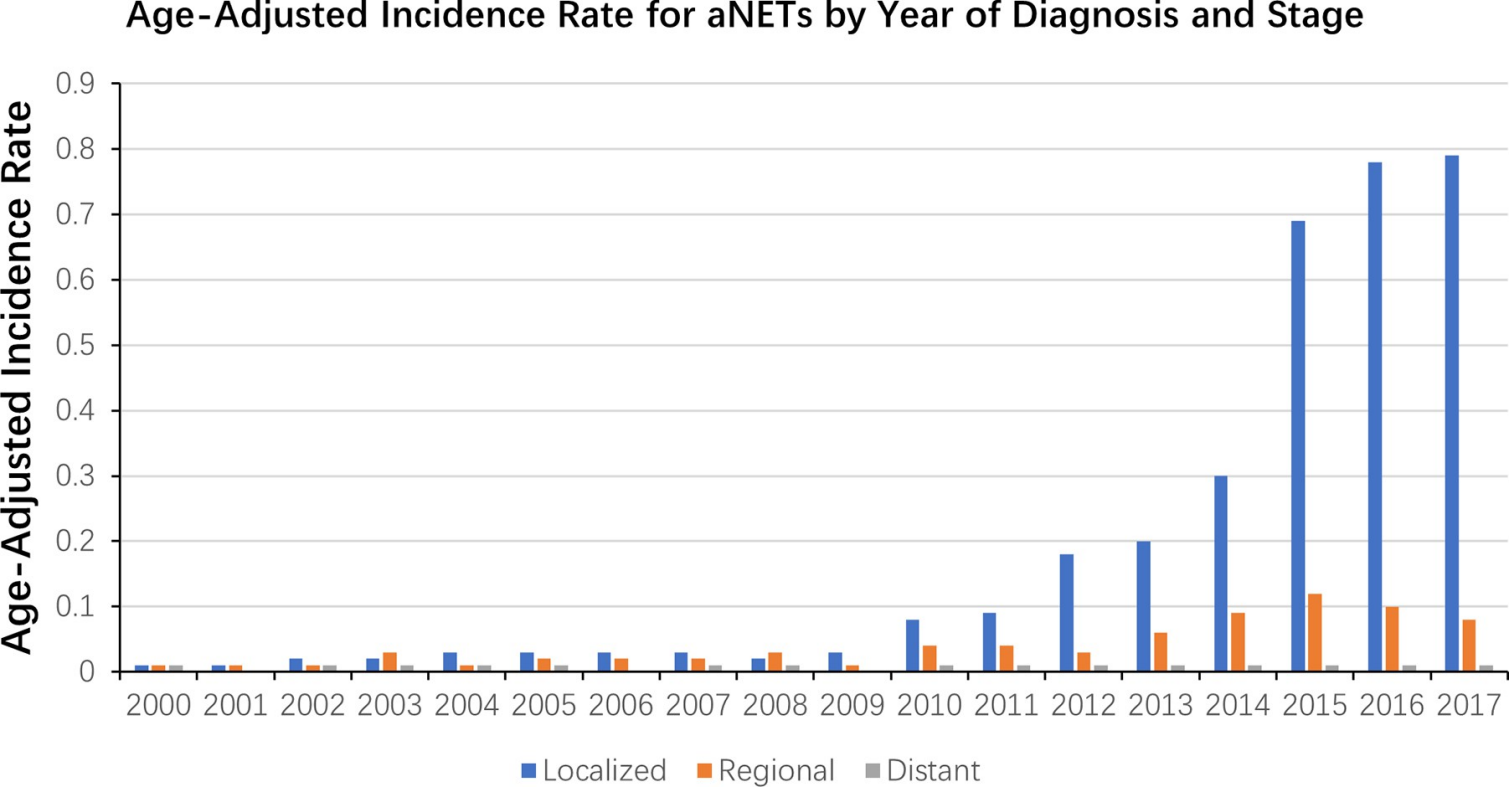
Age-adjusted incidence rate by year of diagnosis and stage in aNETs.

The overall incidence of appendiceal tumors was 0.93 per 100,000 person-years for the period 2000–2017. During this period, the incidence of different pathological types of appendiceal tumors was 0.34 per 100,000 person-years for colonic adenocarcinomas, 0.32 per 100,000 person-years for mucinous adenocarcinomas and 0.25 per 100,000 person-years for aNETs. The annual incidence of appendiceal tumors has increased from 0.47 to 1.72 per 100,000 person-years in the last 20 years. Stratified by pathological type, the annual incidence of colonic adenocarcinoma and mucinous adenocarcinoma remained relatively stable, while the annual incidence of aNETs increased significantly, from 0.03 to 0.90 per 100,000 person-years (**Fig 1**). When analyzed from the stage perspective, the number of patients diagnosed with localized disease has increased significantly in recent years (**Fig 2**), which may have contributed to the increased incidence of aNETs between 2000–2017. The age-adjusted incidence of all appendiceal tumors in white and black patients was 1.02 and 0.70 per 100,000 person-years respectively. The age-adjusted incidence for white and black patients in aNETs were 0.28 and 0.15 per 100,000 person-years. Diagnosis of appendiceal tumors peaks in patients aged 75 to 79 years, with an incidence of 2.58 per 100,000 person-years. The peak diagnosis of aNETs is in patients aged 65 to 69 years, with an incidence of 0.41 per 100,000 person-years (**S1 Fig**).

### Prognosis analysis

**Fig 3:**
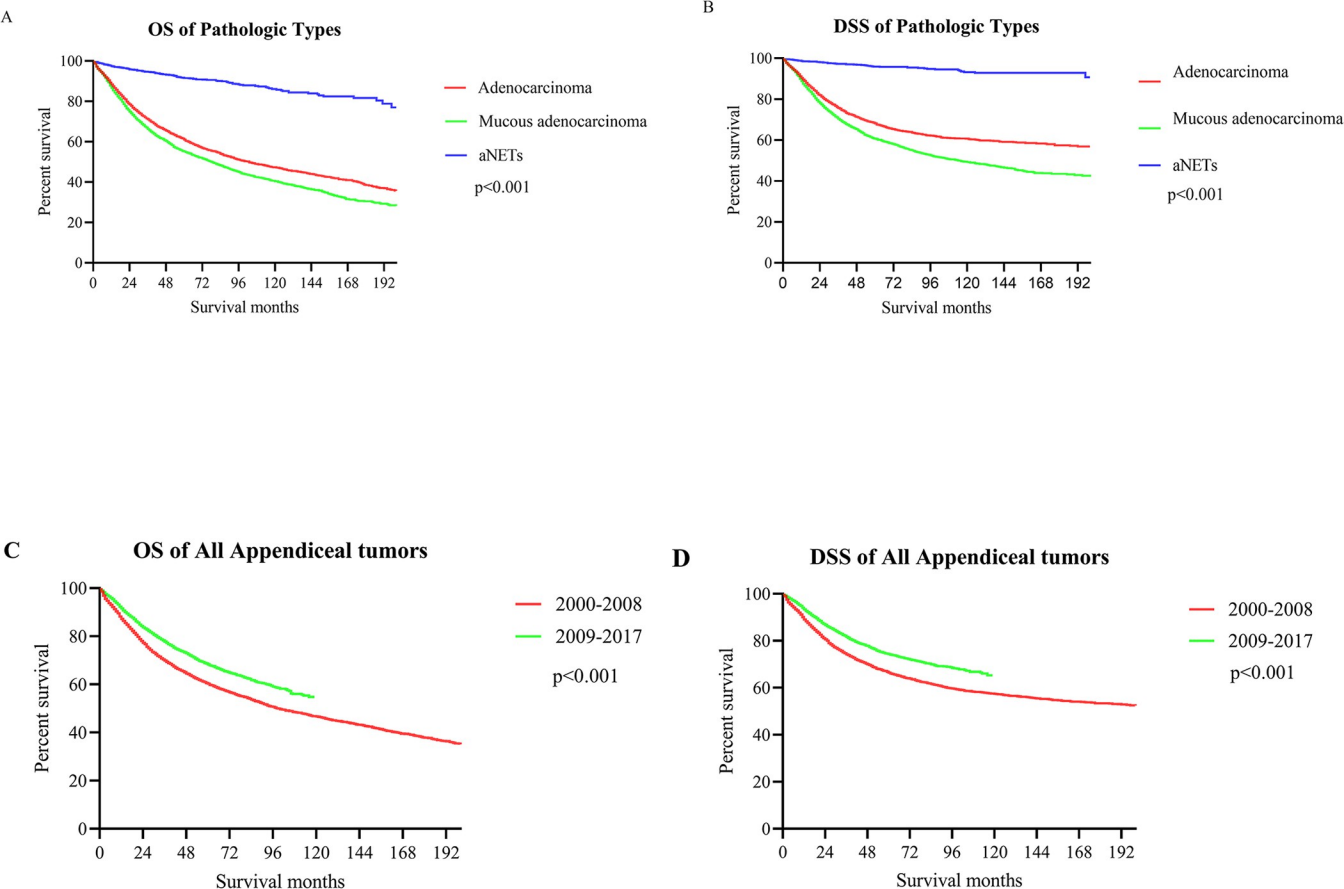
Survival analysis of all patients with appendiceal tumors. (A) The 5-year OS by pathology type; (B) The 5-year DSS by pathology type; (C) The 5-year OS by year of diagnosis; (D) The 5-year DSS by year of diagnosis.

**Fig 4:**
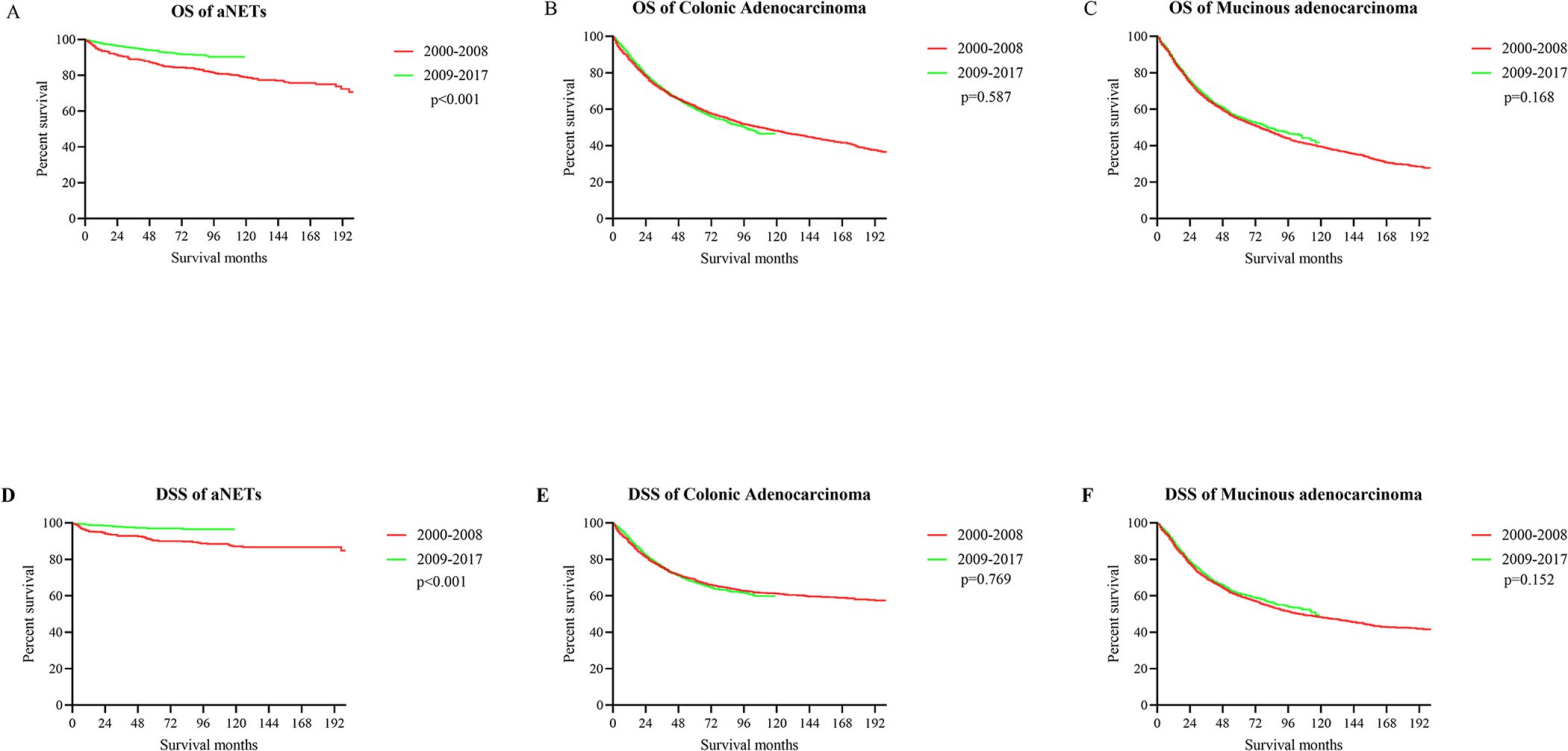
Survival analysis of each pathological type by different years of diagnosis. (A) OS of aNETs; (B) OS of colonic adenocarcinoma; (C) OS of mucinous adenocarcinoma; (D) DSS of aNETs; (E) DSS of colonic adenocarcinoma; (F) DSS of mucinous adenocarcinoma.

The median survival for all patients with appendiceal tumors was 127 months (95% CI [119,134]). The 5-year OS rates by pathology type were 61.09% (colonic adenocarcinomas), 55.20% (mucinous adenocarcinomas) and 89.57% (aNETs), respectively (**Fig 3A**). The 5-year DSS rates were 68.10% (colonic adenocarcinomas), 60.74% (mucinous adenocarcinomas) and 95.90% (aNETs) (Fig 3B). Compared to the period 2000–2008, patients with appendiceal tumors showed a significant improvement in OS (p <0.001; HR = 0.74, 95% CI [0.70, 0.79]) and DSS (p <0.001; HR = 0.74, 95% CI [0.69, 0.79]) during the period 2009–2017 (Fig 3C and 3D). Stratified by pathology type, patients with aNETs likewise showed significantly improved OS from 2009–2017 compared with 2000–2008 (p<0.001; HR = 0.39, 95% CI [0.27, 0.56]) (**Fig 4A**), whereas no such survival improvement was seen in colonic and mucinous adenocarcinomas (**Fig 4B and 4C**). The DSS analysis in each subgroup showed the same trend (Fig 4D–4F).

**Fig 5:**
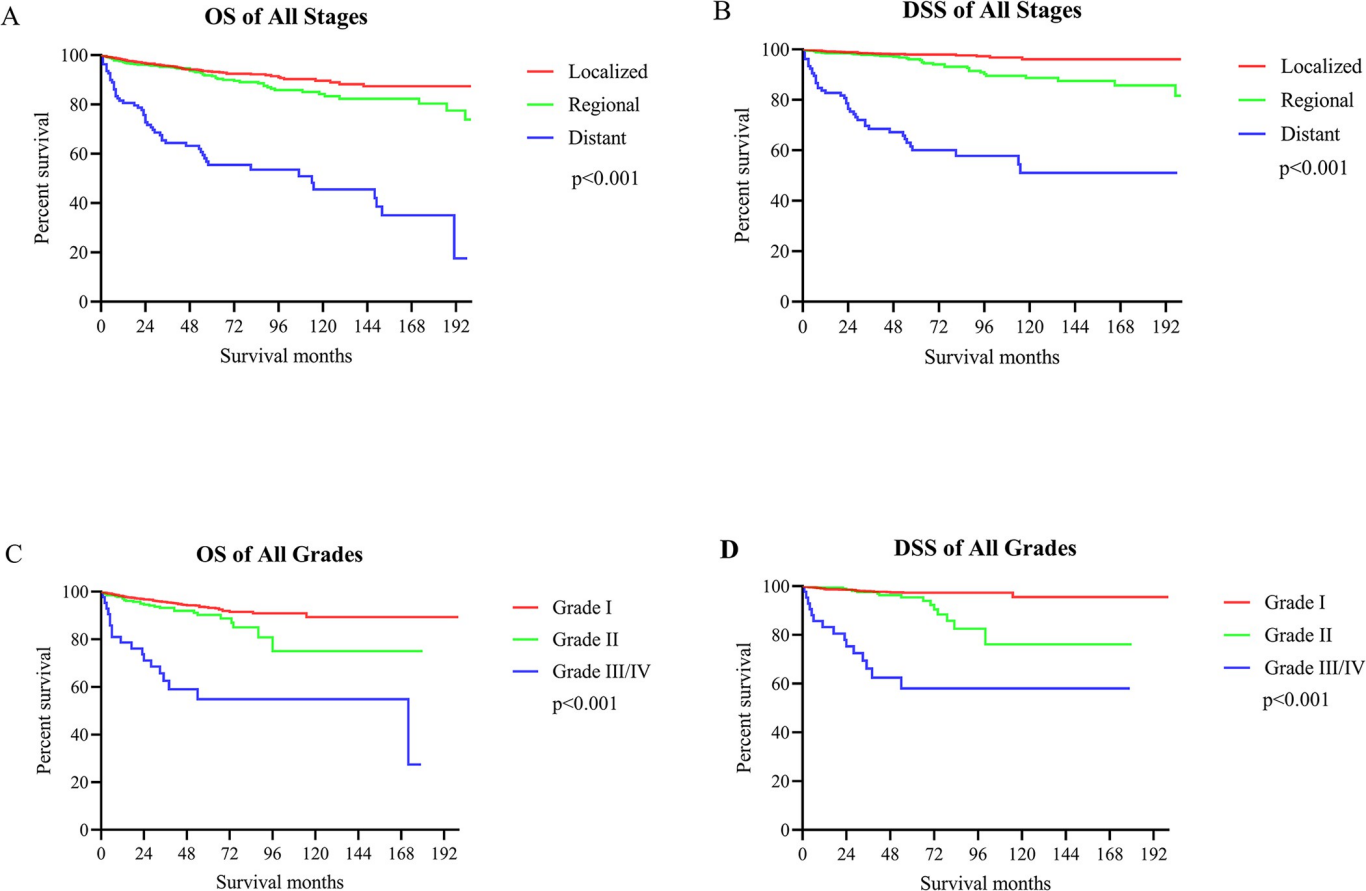
Survival analysis of different tumor stages and grades in aNETs. (A) OS of all stages; (B) DSS of all stages; (C) OS of all grades; (D) DSS of all grades.

**Fig 6:**
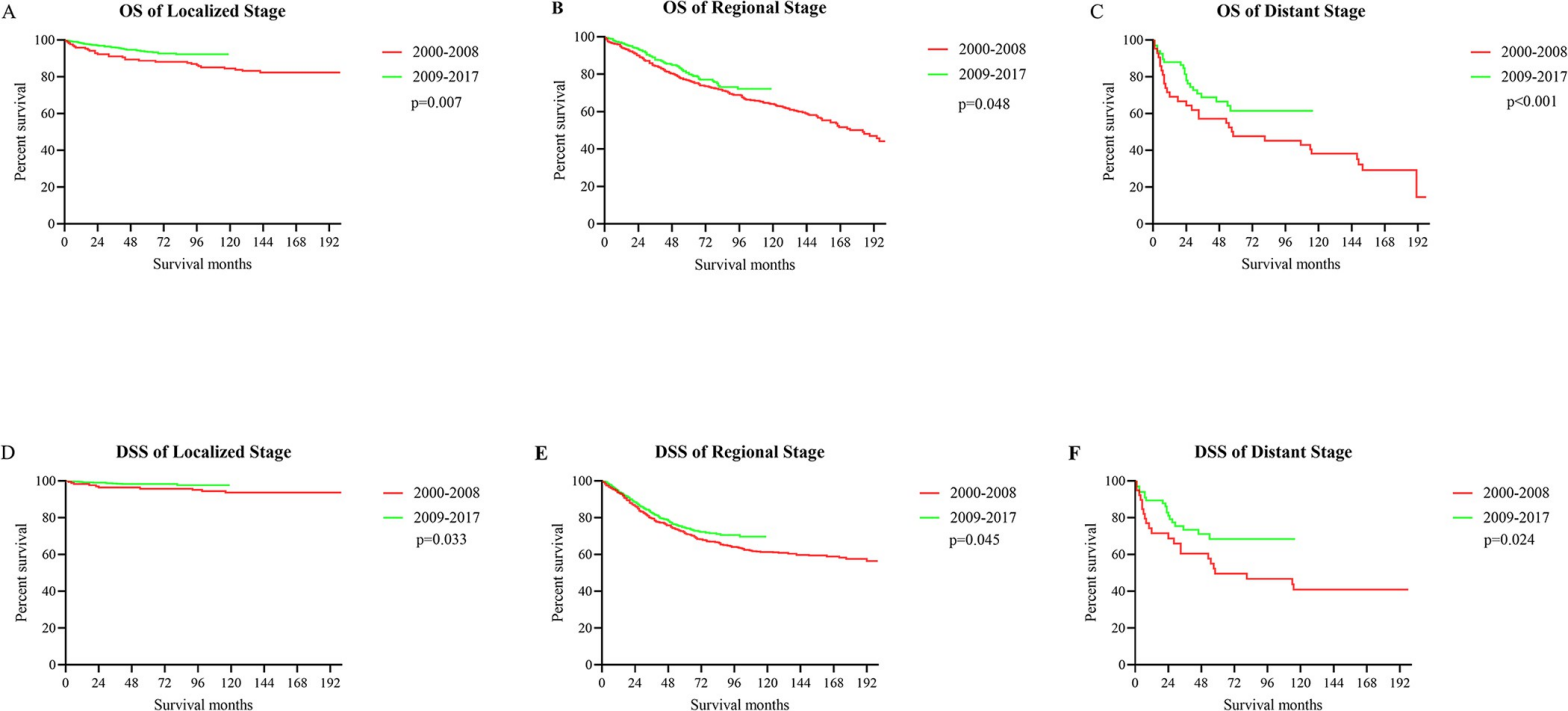
Survival analysis of aNETs with different tumor stages by year of diagnosis. (A) OS of localized stage; (B) OS of regional stage; (C) OS of distant stage; (D) DSS of localized stage; (E) DSS of regional stage; (F) DSS of distant stage.

Further analysis of patients with aNETs revealed that the 5-year OS for local, regional and distant disease were 93.21%, 89.36% and 55.49%, respectively (**Fig 5A**). Correspondingly, the 5-year DSS for each stage of aNETs was 97.96% (localized stage), 90.09% (regional stage) and 60.03% (distant stage), respectively (**Fig 5B**). In addition, the 5-year OS for patients with different tumor grades was 93.22% (Grade I), 87.06% (Grade II) and 58.85% (Grade III/IV) (**Fig 5C**). The 5-year DSS for Grade I, Grade II and Grade III/IV were 97.30%, 93.34% and 58.03%, respectively (**Fig 5D**). It is pleasing to note that this improvement in survival over time was seen at all stages (localized, regional, distant) of aNETs. This is evidenced by the fact that OS and DSS were better in 2009–2017 than in 2000–2008 for all three stages of localized stage (OS: p = 0.007, DSS: p = 0.033), regional stage (OS: p = 0.048, DSS: p = 0.045), and distant stage (OS: p<0.001, DSS: p = 0.024) (**Fig 6**). In univariate Cox proportional risk regression models, younger at diagnosis, more recent year of diagnosis (2009–2017), female gender, married status, localized stage, and low grade were all related to improved OS. Gender, age, time of diagnosis, marital status, tumor grade and stage were also strongly associated with OS in the multivariate Cox proportional risk regression model. In contrast, for DSS, marital status was not statistically associated with prognosis in a multivariate Cox proportional risk regression model (**Table 3**). In addition, surgery may also be a factor influencing the prognosis of aNETs according to the results of our analysis, but further validation is needed.

**Table 3:** Multivariable cox proportional hazards regression for aNETs diagnosed between 2000 and 2017.

	OS				DSS			
Characteristic	Univariate analysis	Multivariate analysis			Univariate analysis	Multivariate analysis		
	P	HR	95%CI	P	P	HR	95%CI	P
Sex	0.001			<0.001	0.013			0.011
Female		Reference	Reference	Reference		Reference	Reference	Reference
Male		1.561	1.215–2.005	<0.001		1.627	1.120–2.365	0.011
Race	0.892				0.674			
White								
Black								
Other and unknown								
Year	<0.001			<0.001	<0.001			0.001
2000–2008		Reference	Reference	Reference		Reference	Reference	Reference
2009–2017		0.523	0.364–0.751	<0.001		0.427	0.260–0.702	0.001
Marital status	0.034			0.016	0.037			0.741
Married		Reference	Reference	Reference		Reference	Reference	Reference
Single		0.680	0.523–0.885	0.004		0.861	0.578–1.281	0.460
Unknown		0.835	0.504–1.383	0.483		1.003	0.465–2.163	0.994
Age	<0.001			<0.001	<0.001			<0.001
44 years or younger		Reference	Reference	Reference		Reference	Reference	Reference
45 to 60 years		5.272	3.325–8.359	<0.001		7.718	3.376–17.648	<0.001
60 years or older		17.464	11.422–26.703	<0.001		22.545	10.227–49.700	<0.001
Grade	0.001			<0.001	<0.001			<0.001
I		Reference	Reference	Reference		Reference	Reference	Reference
II		1.238	0.781–1.961	0.364		0.781	0.349–1.750	0.549
III/IV		3.038	1.786–5.167	<0.001		3.764	1.975–7.174	<0.001
Unknown		0.978	0.701–1.363	0.895		0.833	0.503–1.381	0.479
Stage	<0.001			<0.001	<0.001			<0.001
Localized		Reference	Reference	Reference		Reference	Reference	Reference
Regional		1.287	1.031–1.649	0.049		1.189	0.697–2.029	0.526
Distant		3.440	2.362–5.008	<0.001		7.240	4.323–12.125	<0.001
Unknown		1.190	0.569–2.491	0.644		2.584	1.047–6.379	0.039
Surgery	<0.001			<0.001	<0.001			0.026
No		Reference	Reference	Reference		Reference	Reference	Reference
Yes		0.574	0.422–0.816	<0.001		0.661	0.222–0.895	0.026

## Discussion

Recently, several studies on epidemiology have shown a significant increase in the incidence of appendiceal tumors [12, 13]. This study likewise revealed such a trend and, more importantly, we analyzed the changes in the incidence of appendiceal tumors in different pathological types. From 2000 to 2017, the annual incidence of appendiceal tumors increased from 0.47 to 1.72 per 100,000 person-years. This may be attributed to the marked increase in the incidence of aNETs over the last two decades, with an almost 30-fold increase in annual incidence from 0.03 to 0.9 per 100,000 person-years. In contrast, the annual incidence of the other two adenocarcinoma types of appendiceal tumors is relatively stable. Specifically, the increase in the incidence of aNETs is mainly reflected in the higher proportion of early-stage disease after 2009. In recent years, a better understanding of the pathophysiological manifestations of appendiceal tumors, particularly aNETs, and an improved classification system may have contributed to a marked increase in the incidence of aNETs in appendiceal tumors [14]. In addition, a proportion of patients in the early stages of the disease may be diagnosed incidentally without exhibiting symptoms associated with malignancy [15]. Furthermore, with the increased availability of advanced imaging methods as a clinical assessment tool, we have observed an increasing number of aNETs being diagnosed incidentally in clinical practice, often at an early stage [16].

Despite the significant increase in incidence, patients with appendiceal neoplasms diagnosed between 2009 and 2017 had significantly improved survival rates compared to those diagnosed between 2000 and 2008. Patients with aNETs also show substantial improvements in survival over time. Thus, this study reveals a complex epidemiological phenomenon of increased incidence and decreased mortality in appendiceal tumors. Part of the reason for the improvement in overall patient survival over time may be due to the predominance of the increased incidence of aNETs, which inherently have a better prognosis than other tumor types. Moreover, the increased incidence of grade I tumors may have further enhanced the survival data of aNETs to some extent. While the increasing number of patients being diagnosed in the early stages of the disease may also be one of the reasons, this does not explain the improved survival rates at all stages of aNETs. Therefore, in addition to the reasons for the change in disease stage, we should consider that the improved survival of aNETs may be the result of better treatment strategies. Firstly, the consensus-based guidelines are more detailed and explicit in their recommendations for the surgical treatment of non-metastatic aNETs. Simple appendectomy is an appropriate treatment for lesions < 1 cm, and right hemicolectomy is indicated for patients with lesions > 2 cm [17, 18]. For lesions between 1.0–2.0 cm, factors including age, high-risk features, comorbidities and the possibility of surgical complications need to be discussed and a multidisciplinary approach is necessary [5]. High-risk features include, in particular, deep mid-appendiceal invasion >3 mm, positive lymphovascular/vascular invasion, positive or indistinct cut margins, and high proliferation rates [19]. Over the last 10 years, many treatment options have been developed for metastatic aNETs, including pharmacological treatment with targeted drugs, cytotoxic chemotherapy or somatostatin, and external beam radiotherapy [5, 20, 21]. Long-acting somatostatin analogues were effective in inhibiting tumor growth in randomized phase III trials (CLAIRNET and PROMID) and are used to treat metastatic, well-differentiated aNETs [22, 23]. Cytotoxic chemotherapy is only indicated for aNETs with a heavy tumor burden, high tumor grade and no other treatment options [5]. Based on several clinical trials, Everolimus is approved for several metastatic gastrointestinal NETs, including aNETs [24, 25]. Further research into new predictive biomarkers and disease biology will help to improve our current treatment strategies for aNETs. Moreover, there may be a survival benefit from surgical cytoreduction in well-differentiated patients with metastatic aNETs [5]. Hence, a comprehensive multidisciplinary assessment of many factors including the feasibility of surgery, the status of growth inhibitor availability, tumor burden and histologic grade to select the suitable individualized treatment for each patient is key to prognosis. The above explanations are all hypotheses of this study, and further studies are needed to confirm or explore the real causes of the phenomenon.

However, this improvement in survival over time was not presented in colonic and mucinous adenocarcinoma. In recent years, the treatment of colonic type appendiceal adenocarcinoma basically refers to the treatment standard of colon cancer, which mainly includes right hemicolectomy and chemotherapy [2]. Some studies suggest that current chemotherapies fail to improve the survival rate of patients with appendiceal cancer and are even detrimental to their prognosis [4, 26], which is consistent with the results of our analysis. Therefore, further exploration of specific chemotherapy regimens for appendiceal adenocarcinoma may be needed. Appendiceal mucinous neoplasms are heterogeneous diseases with different malignant potential, a rare and complex disease with different classification and staging systems leading to a great controversy in therapeutic management [27]. The treatment options for appendiceal mucinous tumors have improved in recent years, including cytoreductive surgery, hyperthermic intraperitoneal chemotherapy (HIPEC), and chemotherapy, but overall patient survival has not improved significantly [28]. A possible explanation is that elderly patients account for the majority of appendiceal adenocarcinoma, in this study, patients over 60 years of age accounted for 51.67% of colonic adenocarcinoma and 48.58% of mucinous adenocarcinoma, respectively. The safety and long-term survival of these complex and potentially life-threatening procedures, combination treatments, or systemic chemotherapy in older patients remains controversial [29]. These may be part of the reason why the prognosis of these two appendiceal tumors has not improved over time. However, further studies are needed to determine the exact cause. In addition, this study found that the survival rate of aNETs declined significantly approximately 10 years after diagnosis. Given the risk of late recurrence, the guideline and consensus recommended follow-up strategy includes anatomical imaging of selected patients at 6–12 months post-operatively and annually thereafter for 10 years [30, 31]. These specific follow-up patients include those with incomplete tumor resection, higher grade tumors (G2 or G3), lymph node involvement and/or lymphovascular infiltration [32].

Although there are some previous studies on the incidence of appendiceal tumors [33, 34], the novelty of the present study is that we analyzed not only the incidence but also the change in survival. In particular, the study also analyzed the change in survival over time in subgroups with different stages. Of course, this study is not without its drawbacks due to the properties of retrospective study and limitations of the SEER database. On the one hand, tumor size, mitotic index and the Ki-67 index are crucial for the staging and prognosis of neuroendocrine tumors [35], but these details are not available from the SEER database. Additionally, it is not possible to use SEER data to identify functional tumors because of the unavailability of information on patient comorbidities and other details regarding the functional status of aNETs. Also, the SEER database lacks detailed information on the treatment of patients, such as therapeutic drugs, specific treatment modalities and the number of appendectomies performed in each period, all of which are important for analyzing changes in appendiceal tumor incidence and survival. Despite these limitations, this research provides more comprehensive data to examine trends in the incidence and survival of appendiceal tumors, particularly aNETs, in the United States.

## Conclusion

In the last 20 years, the incidence of appendiceal tumors has shown a significant increase, mainly in aNETs. Additionally, an increasing number of patients are being diagnosed at an early stage of the disease, thus showing a significant migration in stage. We must note that despite an increase in incidence, survival for all stages of aNETs have improved significantly.

## Supporting information

S1 Fig (TIF)

S1 File Age-adjusted incidence rates of all patients. (CSV)

S2 File Age-adjusted incidence rates of aNETs. (CSV)

S3 File Age-adjusted incidence rates of colonic adenocarcinoma. (CSV)

S4 File Age-adjusted incidence rates of mucinous adenocarcinoma. (CSV)

S5 File Raw data. (XLSX)

## Abbreviations

SEER: Surveillance, Epidemiological, and End Results
aNETs: appendiceal neuroendocrine tumors
OS: overall survival
DSS: disease-specific surviva
CI: : confidence interval
HR: hazard rati
