# Supplementary figures and images for: Incidence trends and survival analysis of appendiceal tumors in the United States: Primarily changes in appendiceal neuroendocrine tumors
Source: PLoS One. 2023 Nov 13;18(11):e0294153. doi: 10.1371/journal.pone.0294153 (PMC10642837; doi:10.1371/journal.pone.0294153)

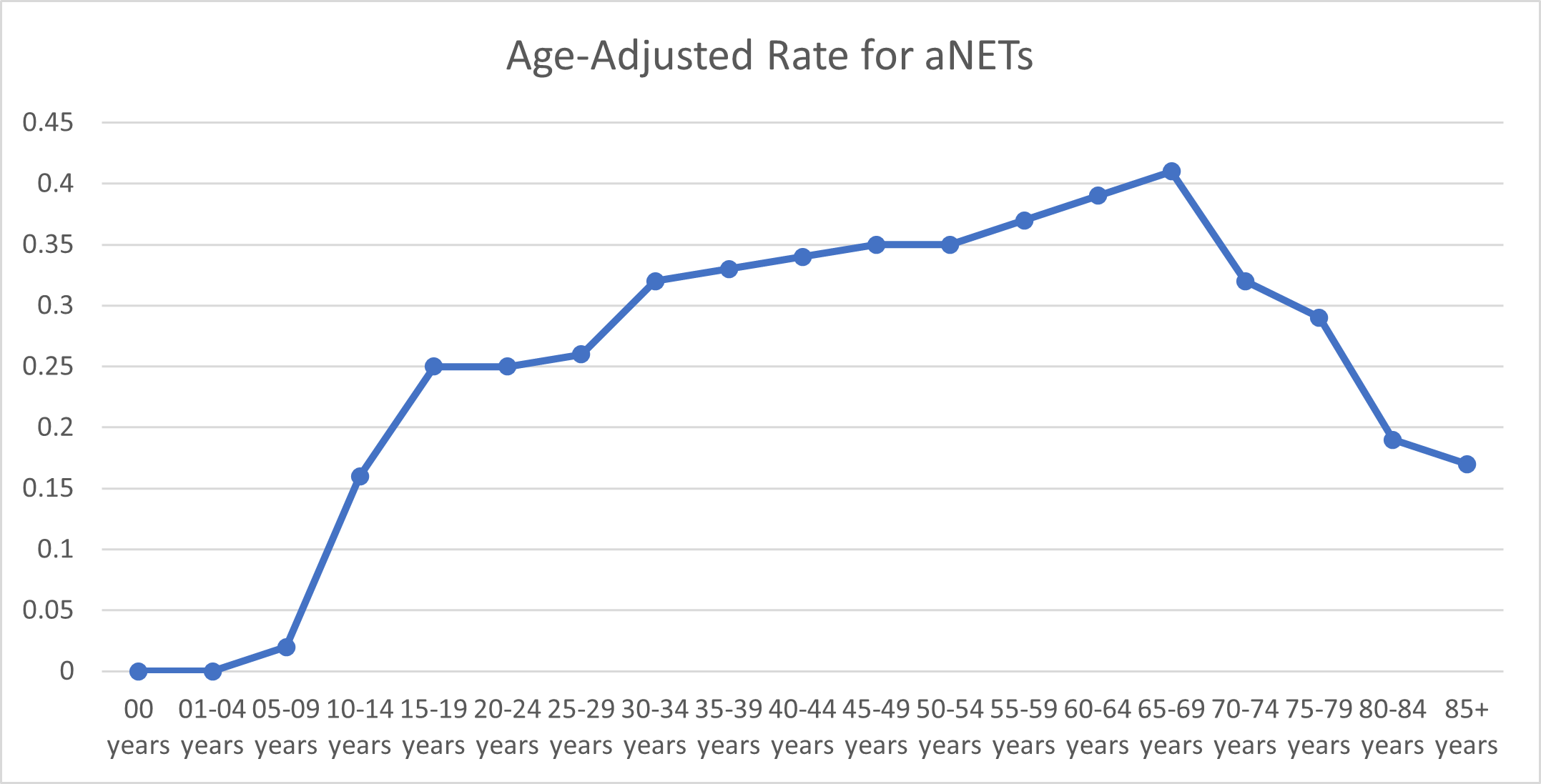

Supplement: S1 Fig — (TIF) [file pone.0294153.s001.tif]
